# Climate and Edaphic Factors Partly Explain Leaf Litterfall Rates of Species From Tropical Semi‐Deciduous Ecosystems

**DOI:** 10.1002/ece3.74224

**Published:** 2026-08-20

**Authors:** Ashehad A. Ali, Anuj T. Magar

**Affiliations:** ^1^ Department of Bioclimatology University of Göttingen Göttingen Germany; ^2^ Bioeconomy Science Institute – AgResearch Group Lincoln New Zealand

**Keywords:** global synthesis, litterfall, phenology, semi‐deciduous, tropics

## Abstract

Carbon cycling in semi‐deciduous ecosystems is difficult to observe because plant species shed leaves for only a short period (4–6 weeks), and some remain dormant for only a few weeks. These short phenological phases limit ecosystem studies. To our knowledge, no database currently describes spatial and temporal patterns of litterfall in semi‐deciduous ecosystems. Therefore, this study aimed to identify factors associated with leaf litterfall rates in these ecosystems. We compiled leaf litterfall data from 31 global semi‐deciduous sites in the literature and collected climatic data (precipitation, air temperature, vapor pressure deficit, radiation, daylength) and edaphic data (available water storage capacity, top‐soil bulk density, clay cation exchange capacity, clay and sand fractions, pH, and soil carbon). Models of linear mixed effects (LMEM) and random forest were used to examine climatic and soil controls on litterfall rates. Radiation, daylength, rainfall, and soil pH explained 51% of the variation in litterfall rates across the data set that was used for calibration. All the predictors were negatively correlated with litterfall. The LMEM and random forest model revealed consistent key predictors of the litterfall. When the developed model was applied at independent sites, the explanatory power of the model was 24%, suggesting the need to include biotic factors such as species composition, vegetation structure, and functional traits in the model. Our synthesized datasets can be used to calibrate litterfall rates that are predicted using process‐based models, such as land surface models.

## Graphical abstract

Semi‐deciduous ecosystems have short leaf‐shedding periods, making carbon cycling and litterfall patterns difficult to study. This study compiled data from 31 global sites and used regression models to examine how climate and soil factors influence leaf litterfall rates. Results show that both climatic factors (radiation, daylength, rainfall) and soil properties (soil pH) partially explain litterfall variation.

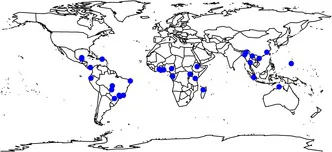

## 1 Introduction

Semi‐deciduous ecosystems shed their foliage for a short period of time (Monasterio and Sarmiento 1976; De Weirdt et al. 2012) compared to tropical fully deciduous ecosystems (TFDEs), that is, fully deciduous ecosystems can be leafless for 3–4 months of the year whereas semi‐deciduous ecosystems are leafless for only 4–6 weeks of the year. Semi‐deciduous ecosystems form the transition between fully deciduous and evergreen ecosystems.

Semi‐deciduous tree species appear across global tropical regions, including in Australia (Williams et al. 1997), Ghana (Dawoe et al. 2010), Indonesia (Kotowska et al. 2016), Thailand (Takahashi et al. 2012), Panama (Cavelier et al. 1999), Brazil (Haase and Hirooka 1998; Sanches et al. 2008), and Mexico (Aryal et al. 2015). These tree species show up in diverse settings like native savannas, natural seasonal forests, and managed agricultural plantations. However, a comprehensive study of litterfall dynamics of these ecosystems is scarce even though these ecosystems cover relatively large territories in the tropics. Compared to TFDEs, semi‐deciduous systems retain a significant portion of their foliage during the dry season and exhibit less variation in greenness throughout the year.

Leaf litterfall is a key driver of soil nutrient cycling in the tropics. It is also a marker of seasonal change (leaf shedding) in the tropics, particularly in regions where there are distinct wet and dry seasons. Leaf litterfall peaks as the water stress, vapor pressure deficit, and radiation increase while air temperature decreases (Reich 1995). By shedding leaves, plants prevent losing a lot of water from soil. The shedding of leaves enables the plant to obtain more efficient foliage.

There have been numerous studies on TFDEs that explored temporal dynamics of litterfall. Litterfall from TFDEs was recorded highest at the beginning of dry months and the sites with high soil moisture content showed maximum litterfall 2 months later (Martinez‐Yrizar and Sarukhan 1990). The litterfall pattern in secondary mixed deciduous forest in Thailand peaked in the dry period and dipped in the wet period; however, it was driven by other factors such as species composition, successional stage and land use type (Podong et al. 2013). In the study carried out in Australian Savanna, two out of three tropical fully deciduous species leaf fall started in the transition from wet to dry period, which coincided with the decrease in soil moisture and relative humidity, suggesting litterfall's association with atmospheric and soil moisture (Williams et al. 1997).

However, there have been only a handful of studies on TFDEs that explored spatial dynamics of litterfall. In one study, the spatial litterfall pattern in tropical and subtropical forests of Taiwan was negatively correlated to the elevation of the site but showed no relation with the slope (Wang et al. 2016). A second study in a tropical fully deciduous forest in Mexico showed that the site with gentle slope had more moisture content than that on steeper slope, and litterfall on sites with low soil moisture content starts soon after the end of wet season, but litterfall is delayed on sites with high moisture content (Martinez‐Yrizar and Sarukhan 1990).

Like TFDEs, semi‐deciduous ecosystems show similar litterfall pattern, that is, peak in the dry season and less in the wet season (Morellato 1992). In Indonesia total aboveground litterfall of rubber trees was significantly higher in the dry period than the wet period (Kotowska et al. 2016). A study on cocoa ecosystems in Ghana reported that the litterfall rates in a newly converted cocoa plantation were lower compared to secondary forests and other mature plantation (Dawoe et al. 2010). In general, compared to TFDEs, litterfall rates and their dynamics are less studied from semi‐deciduous ecosystems.

Therefore, this study aims to: combine monthly litterfall data from different sources and (1) examine if annual litterfall rates vary among grouped sites (e.g., southern hemisphere sites vs. northern hemisphere sites), (2) examine if dry‐ and wet‐season litterfall rates vary spatially, and (3) investigate the relationship between changing daylength and rainfall amounts.

There are a few detailed measurements that directly relate to the phenology of semi‐deciduous forests. For example, in Costa Rica, Daubenmire (1972) has characterized phenology of semi‐deciduous forests by making weekly observations of leaf senescence over a year, whereby peak litterfall rates coincided with the middle of the dry season for some plant species and most of these species remained leafless late in the dry season. We hypothesize (H1) that peak litterfall occurs either in the middle of the dry season or toward the end of the dry season. Borchert et al. (2002) also studied phenology of tropical semi‐deciduous forests from Costa Rica and found that decreasing daylength could be triggering leaf abscission and so leaves were being shed rapidly. Therefore, we hypothesize (H2) that decreasing daylength increases litterfall rates. Njoku (1964) has shown experimentally that daylength control over phenology is important at 10° latitude in Africa, and Lawton and Akpan (1968) has provided evidence of its effectivity at 7°26′.

In the context of drought‐induced phenology, Chave et al. (2010) examined seasonal patterns of litterfall and rainfall of tropical forests from South America at a regional level. They found a weak but significant correlation between litterfall seasonality and rainfall seasonality. In the case of semi‐deciduous forests from the Yucatan Peninsula, Mexico, Aryal et al. (2015) found a significant negative correlation between monthly litterfall and monthly rainfall. Therefore, we hypothesize (H3) that decreasing rainfall would increase litterfall.

## 2 Methodology

### 2.1 Study Area

In this study, our aim was to look into research articles that presented leaf litterfall data. Therefore, we used the free web search engines “Google Scholar” and “Web of Science.” We searched the literature between 10th September and 15th December 2021, then for a couple of weeks in February 2022, and recently from 6th to 17th April 2026. We searched for research articles using these terms: “semi‐deciduous forest,” “tropics,” “semi‐evergreen,” “leaf litterfall,” or “brevi‐deciduous.” We focused on semi‐deciduous forest ecosystems that included both natural forests and monoculture matured plantations from the tropics. When we performed the literature search, we did not include non‐English papers. We limited our search to titles, abstracts, and keywords when we performed the literature search. We used manual cross‐checking and removed the exact matches of duplicated studies. If the study in the duplicated sites performed their measurement in a different year, then we included that study.

After downloading the research articles, we went over each article's methodology section and checked that the ecosystem was indeed semi‐deciduous or semi‐evergreen. Then for every article, we looked at whether the researchers reported the values of monthly litterfall rates and their unit. Minimum requirements for inclusion in this study were that leaf litter rates were provided for at least one growing season. Then we digitized the data from each of the research articles using a free digitizing software “Engauge Digitizer”: http://markummitchell.github.io/engauge‐digitizer. In general, there are studies that have digitized data from figures or graphs (Walker et al. 2014; Ali et al. 2015).

We ensured that the publication included essential metadata such as names of dominant plant species, site location, soil type, and sampling time. We considered publications that reported leaf litterfall on either monthly or fortnightly scales. For fortnightly scales, we aggregated it to monthly. We noted that the reported litterfall rate was in different units: “Mg/ha/mon,” “kg/ha/mon,” “tonne/ha/0.5mon,” “g/m2/mon.” To ensure consistency across all sites and have a standard unit, we converted all litterfall rate units in this study to “g/m2/mon.” A site indicates the location of an ecosystem. Every site variable is unique in terms of location (latitude and longitude). If the two sites have the same latitude and longitude values, they are still different in terms of lowland or upland locations. For the definition of a region, please see the second paragraph in the Section 2.3. Our final dataset included 18 sites from the Northern Hemisphere and 13 sites from the Southern Hemisphere (see Table 1). Our data spanned from 1970 to 2024.

**TABLE 1:** Sources of litterfall data gathered for analyses in this study include information about the location and data collection period.

Country	Site	Year	Sample size (*n*)	Latitude	Longitude	References
Ghana	Ashanti region	2006–2007	24	7.25	−2.86	Dawoe et al. (2010)
Thailand	Kanchanaburi Province	1997–1998	24	14.58	98.87	Takahashi et al. (2012)
Indonesia	Jambi Province	2013–2014	12	−1.78	102.81	Kotowska et al. (2016)
Brazil	Cuiaba	1994–1995	14	−15.70	−56.10	Haase and Hirooka (1998)
Brazil	Sinop	2001–2007	72	−11.41	−55.32	Sanches et al. (2008)
Panama	Barro Colorado Island	1987–1989	18	9.15	−79.85	Cavelier et al. (1999)
Ghana	Kade	1970–1972	26	6.15	−0.92	John (1973)
Brazil	São Paulo (Upland)	1985–1986	12	−25.45	−52.91	Morellato (1992)
Brazil	Parana State	2007–2010	48	−23.18	−46.87	Dick and Schumacher (2020)
Mexico	Yucatán Peninsula	2012–2014	24	18.62	−89.55	Aryal et al. (2015)
Brazil	São Paulo (Lowland)	1985–1986	12	−25.45	−52.91	Morellato (1992)
Brazil	São Paulo	2016–2017	12	−22.0	−47.67	Bachega and Tanaka (2025)
Tanzania	Mt. Kilimanjaro	2012–2013	15	−3.08	37.35	Becker et al. (2015)
Benin	Pahou	2016–2017	12	6.42	2.18	Kolimedje et al. (2021)
Gabon	Ogooué Ivindo	2011–2012	12	−0.85	12.25	Iponga et al. (2020)
India	Nagaland	2016–2017	12	26.1	94.25	Leishangthem and Singh (2021)
Brazil	Minas Gerais	2005–2006	12	−21.95	−42.90	Machado et al. (2018)
Uganda	Budongo Forest Reserve	2018–2019	12	1.73	31.53	Manu et al. (2024)
Mexico	Yucatán Peninsula	2014	12	20.08	−89.53	Morffi‐Mestre et al. (2020)
Guam	Karst Forest	2013	12	13.52	144.82	Marler and Cruz (2022)
Nigeria	Ogun State	2004–2005	12	7.97	11.03	Oladoye et al. (2010)
Madagascar	Badrala	2008–2009	12	−15.7	46.25	Pichon et al. (2015)
Equador	Cajas National Park	2014–2015	12	−2.85	−79.22	Pinos et al. (2017)
Puerto Rico	El Tallonal	2008–2009	12	18.41	−66.73	da Silva (2015)
Brazil	Northeastern Brazil	2016–2017	12	−5.9	−35.17	Silva et al. (2020)
Ethiopia	Kewet district	2017	12	10.18	39.45	Tesfay et al. (2020)
China	Fujian	1999	12	26.19	117.43	Yang et al. (2004)
Australia	Darwin	1992–1994	28	−12.4	131.1	Williams et al. (1997)
India	Mizoram	2015–2016	12	23.45	92.75	Singh et al. (2025)
China	Xishuangbanna Prefecture	2017–2019	36	21.91	101.25	Yuan et al. (2024)
China	Hainan Island	2023–2024	12	19.3	108.92	Zhang et al. (2025)

*Note:* Sample size (*n*) indicates the monthly measurement values of litterfall rates.

**FIGURE 1:**
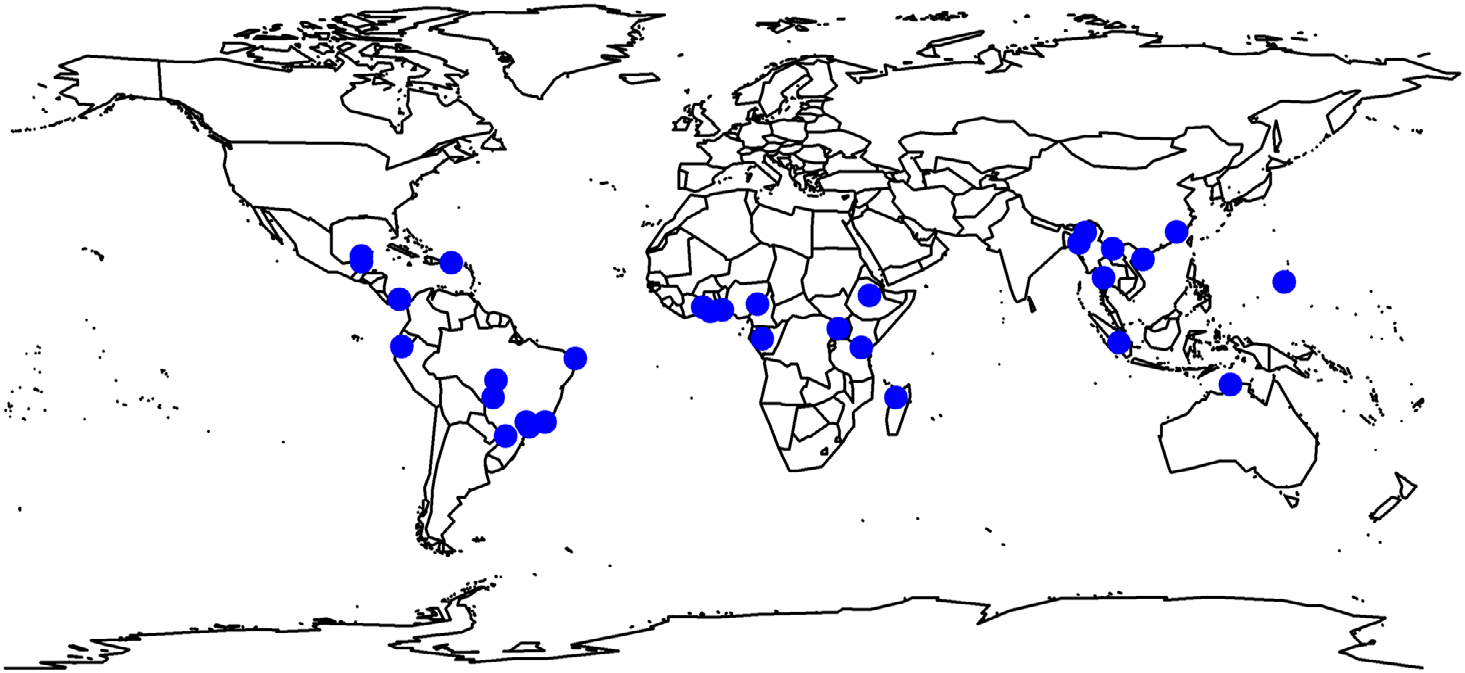
World map showing the location of the sites of the collected data.

Our dataset included five plantation sites and 26 forest sites. With regards to different continents, our dataset contains nine sites from Asia and Oceania, nine from Africa, and 13 from Central and South America (Figure 1). The blue points in the figure indicate the location of sites on the world map (Figure 1).

### 2.2 Climate and Soil Data

Climatic data such as precipitation, air temperature, air pressure, wind speed, dew point temperature, and global radiation corresponding to the location and the time of litterfall data collection period were obtained from ERA5, which is a reanalysis product based on the Integrated Forecast System of the European Centre for Medium‐Range Weather Forecasts (ECMWF‐IFS; Hersbach et al. 2020). We obtained ERA5 with the DOI: https://doi.org/10.24381/cds.adbb2d47 and accessed it on April 15–20, 2026. ERA5 dataset has a spatial resolution of 0.25° and a temporal resolution of hourly. We aggregated the ERA5 data to get monthly means or sums. Relative humidity (RH) was obtained from air temperature and dew point temperature. Saturated vapor pressure (SVP) was calculated using air temperature, and SVP and RH were used to calculate vapor pressure deficit. The duration of daylight (day length) was calculated using latitude, longitude, and Julian Day.

The study by Brosed et al. (2022) indicated that 0.25° data could be suitable for mapping large‐scale patterns (such as regional or global) of leaf litter decomposition across climatic zones. To study litterfall patterns of tropical forests in Taiwan, Wang et al. (2016) used precipitation data from Tropical Rainfall Measuring Mission that had a 0.25° spatial data and combined it with field litterfall. We acknowledge that for studying individual sites, 0.25° resolution data may not represent the actual temperature or moisture. However, our study is on the global scale so using 0.25° climate data could be appropriate.

Although the research articles that emerged from Google Scholar or Web of Science provided the values of litterfall rates, they barely reported data related to soil. To have a consistent data source for soil across all sites, we accessed the Regridded Harmonized World Soil Database (HWSD) (Wieder et al. 2014). The HWSD data are at 0.05‐degree spatial resolution for the nominal year 2000. The soil data included available water holding capacity, topsoil bulk density, cation exchange capacity, topsoil clay fraction, topsoil sand fraction, topsoil pH (in H_2_O), and area‐weighted topsoil organic carbon content.

We note that the HWSD data are statistic and at a spatial resolution of 0.05°. There is a mismatch in spatial and temporal resolutions. The 0.05° HWSD data are much more detailed and have good soil estimates. The 0.25° climate pixel may average out variations caused by topography such as elevation and aspect that dictate actual leaf‐litter fall, but this might be more inappropriate for studying small scale plots. It is of course better to dynamically or statistically downscale 0.25° climate. During some stage of downscaling, it will require integration or validation with local climate data. There is however limited local scale climate data. For regional‐to‐continental scale analyses of leaf litter, we think both datasets are reasonable to use, but they may not be suitable for local‐scale or microclimate‐dependent studies. To quantify the contribution of climate to leaf traits at the global scale, some studies (e.g., Maire et al. 2015) have used 0.05° HWSD data and linked it to similar spatial scale climate data from the 0.17° Climatic Research Unit (CRU) CL2.0 dataset.

The climate and litterfall vary but soil is static and nominal for 2000. We assumed that properties like soil texture, soil pH, or initial carbon levels were static and thus remained constant between the HWSD 2000 year and the litterfall/climate study years. We think it is a reasonable assumption. Some studies have made similar assumptions when looking at how global leaf traits covary with climate and soil (Maire et al. 2015; Xing et al. 2021). We did not focus on dynamic soil properties such as soil moisture, land‐use changes, soil erosion, or organic carbon change over time, which would have been problematic.

### 2.3 Data Categorizing

After combining the climate and soil data, we identified the months that corresponded to the different seasons. This was done to address the first hypothesis (H1) – peak litterfall occurs either in the middle of the dry season or toward the end of the dry season. The identification of the months was done by looking into relevant studies of each site. We considered five seasons: Early‐Dry season, Mid‐Dry season, Late‐Dry season, Early‐Rain season, and Rainy season. These are defined in the Excel spreadsheet and in Table S1.

Some of the sites seemed to be nested in regions, so we calculated distance using the “goesphere” package in R by specifying the latitude and longitude and using a nested for loop. We checked the sites that were within 200 km. Note that this distance is equivalent to the distance between Los Angeles and San Diego, USA. We were able to identify 27 Regions.

To determine the sites that could be used for developing the model (“calibrating the model”) and the remaining sites for validating or testing the model, we used a random sampling approach without replacement, whereby we used 75% of the sites for calibrating the model and 25% of the sites for validating the model. A map showing the separation of the sites into two datasets, one for calibrating the model and the other for validating the model is shown in Figure S1.

Since litterfall is also a delayed response to environmental variables and often caused by preceding climatic conditions, we considered three dataset groupings; climate measurements from the same month as litterfall measurements, climate measurements lagged by 1‐month and climate measurements lagged by 2 months. The lagged climate variables were matched by the calendar month within each site where climate variables were obtained from a specific prior month. The missing first‐month or first‐two‐month observations were considered null values, that is, “NA.” The above methods enabled us to test for the lagged effects of climate on litterfall too.

### 2.4 Linear Mixed Effects Model

Our dataset has a hierarchical structure where the sites are located within regions. Therefore, to explore environmental controls on leaf litterfall rates, we opted to use a linear mixed effects model (LMEM). In LMEM, the fixed‐effect term was the climate or the soil variable allocated to each site; site and region were considered as random intercepts. To account for repeated measures data where observations closer in time are more correlated than those further apart, we considered an autocorrelation term in the LMEM using the “corAR1” function. The AR1 autocorrelation was applied within site. To identify the most important variables for explaining the litterfall, we used the “buildmer” package in R, which involves a stepwise elimination technique. All potential predictors (all variables that were log transformed (see the Section 2.7) but not litterfall) and random effects were specified in the model. Log of litterfall was the response variable. We specified the direction as “backward” and set the criteria option to “Akaike's Information Criterion” (AIC). The model then performs stepwise elimination by minimizing AIC. The buildmer tool bas been used when trying to establish links between herbivore arthropods and leaf traits (Schön et al. 2023). To our knowledge, Walker et al. (2014) employed a backward stepwise AIC‐based option in a linear mixed effects model when examining relationship between leaf photosynthesis and leaf traits at the global scale.

We used the above stepwise elimination method for three different data sets: climate measurements from the same month as litterfall measurements, climate measurements lagged by 1‐month and climate measurements lagged by 2 months. Because climatic variables could be correlated, we examined multicollinearity among the significant predictors by examining variance inflation factors. The LMEM that was obtained via the elimination method was further reduced (i.e., predictors were reduced) based on the significance of the *p*‐values and correlations among the fixed effects.

### 2.5 Advance Modeling

To complement the final LMEM and compare it with other modeling methods, we used an advanced analytical framework that integrated correlation analysis and robust modeling methods. Specifically, we combined Pearson correlation and random forest model. The Pearson correlation accounts for the linear relationships, and the random forest model handles non‐linear interactions. There were two main steps:

- Using all potential predictors and setting the Pearson option of correlation and cut‐off frequency to 0.75, we removed the highly correlated variables.
- Then we ran the random forest model by using the filtered predictors, litterfall as the response variable and setting ntree as “5000” and importance to “True”.

The above steps allowed us to examine the explanatory power of the model, determine how much the model's accuracy drops if a predictor's values are randomly shuffled, and identify the most important predictors.

### 2.6 Vegetation Structure Data

We also obtained some of the data related to vegetation structure (stand density, canopy height, basal area, dbh, leaf biomass and leaf mass per area) from their respective publications. Unfortunately, each of these variables were available for between 20% and 50% of the sites (as can be seen in Table S2). Stand density data was from 14 sites, while DBH (Diameter at Breast Height) was from 12 sites (Table S2). Basal Area was reported across 9 different sites, and Canopy Height data was from 7 sites. LMA (Leaf Mass per Area) was limited to 2 sites, and Leaf biomass was only from 1 site.

Since stand density was available for about 50% of the sites, we filtered data for the sites where stand density was available and then considered stand density in the linear mixed effects model. Here, the predictor was only stand density. The prediction power of stand density for litterfall was little (*r* ^2^ = 0.05), so we did not consider stand density ultimately in the derived model.

To fill in some of the missing data, we tried to obtain canopy height data from ‘forest data’ in R using the ‘terra’ package by providing site specific latitude and longitude values. After obtaining the data and comparing it with the known estimates from some of the site publications, we noted a large discrepancy between the two datasets—one likely reason could be that the ‘forest data’ has a fine spatial resolution (~10 m). Therefore, we did not fill in the canopy height data.

### 2.7 Statistical Analysis

The climatic, soil and other variables together with definitions and units are listed in Table S1. We considered the following variables:

“T_Mean,” “T_max,” “T_min,” “RH_Mean,” “RH_max,” “RH_min,” “VPD_Mean,” “VPD_max,” “VPD_min,” “Rad_Mean,” “Rain_sum,” “AP_Mean,” “DayL_Mean,” “WS_Mean,” “litterfall,” “SandF,” “ClayF,” “SiltF,” “Water_Storage,” “Water_Storage_mm,” “Bulk_density,” “Clay_cation_exchange,” “Soil_PH,” “Carbon_content,” “Soil_Organic_Carbon.”

Prior to the log transformation, visual checks were made to see if each of these variables were approximately normally distributed. We noted that they were skewed to the right. To test if the variables were normally distributed or not, we tested the above variables using Shapiro–Wilk test (Halbritter et al. 2025) one variable at a time. All the *p*‐values were less than 5%, indicating that the data was not normally distributed. Then we performed the log transformation. Scaling the data such as taking the log transformation is a common approach used in leaf trait ecology (e.g., Leishman et al. 2007; Walker et al. 2014; Maire et al. 2015). To sum up, we log‐transformed the variables to address skewness and non‐constant variance. After model fitting, we examined the resulting residuals for normality checks.

In this study, we used Tukey's test to compare all possible pairs of means for a set of categories (i.e., when we group sites). To test the first hypothesis, that is, if the peak litterfall occurs in the middle of the dry season or toward the end of the dry season for our global dataset, a two‐sample paired *t*‐test was used. To test the second (decreasing daylength increases litterfall rates) and the third hypotheses (decreasing rainfall increases litterfall rates), we used the standardized major axis slopes (“smatr”) method as used in other studies (e.g., Leishman et al. 2007). Because both variables can potentially contain measurement errors, we chose to use SMA. An SMA fit minimizes sums of squares in *X* and *Y* dimensions simultaneously. It gives the slope of the first component from a principal components analysis calculated from a correlation matrix (Wright et al. 2005).

To examine the performance of the linear mixed effects model, we looked at (i) the coefficient of determination (*R* ^2^ value) and *p*‐values, (ii) the residual diagnostic plots, and (iii) performed multicollinearity tests. In the case of the random forest model, we used the Pearson correlation.

All statistical analyses were performed in R version 4.5.3. Linear mixed‐effects models (LMEM) were fitted using the *lme4* package. We checked for collinearity among predictors by calculating variance inflation factors (VIF) using the *vif* function in the car package. Marginal and conditional *r* ^2^ values for the LMEMs were extracted using the *MuMIn* package. The random forest regression models were implemented via the *randomForest* package. Additional analyses were also performed in R using *terra* for spatial data, *smatr* for allometric fittings, and temporal/spatial autocorrelation in model residuals was accounted for and assessed using *buildmer* utilizing *nlme* (*corAR1*) structures for mixed models.

## 3 Results

### 3.1 Changes in Leaf Litterfall Rates

**FIGURE 2:**
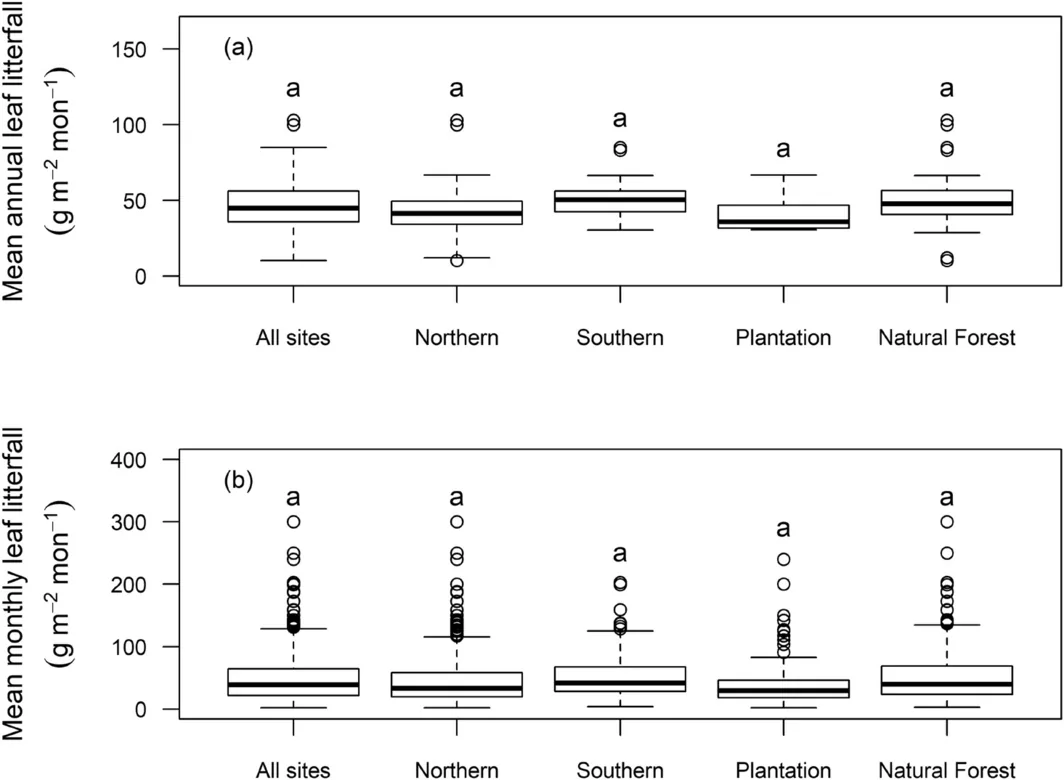
Distribution of (a) mean annual leaf litterfall rates (g m^−2^ mon^−1^) and (b) mean monthly leaf litterfall rates (g m^−2^ mon^−1^) across different groupings of sites; all sites, northern hemisphere, southern hemisphere, plantations and natural forests. Same letters on top of each box plot within a subfigure indicate that the means are not significantly different between the different groupings of sites. The means were compared using the Tukey test (*p*‐value < 0.05).

There was a noticeable difference in the distribution of mean annual litterfall rates (MALR) among grouped ecosystems, where some grouped ecosystems had unusually high MALR values (Figure 2a). Plantations exhibited a narrow distribution of MALR (Figure 2a). The MALR did not vary significantly among grouped ecosystems (Figure 2a). All sites had a median value of MALR as 44.9 g m^−2^ mon^−1^ (Figure 2a). The median value of MALR for Natural Forest (47.8 g m^−2^ mon^−1^) was higher than that of Plantations (35.8 g m^−2^ mon^−1^) but it was insignificant (Figure 2a). The difference in median value of MALR between Northern and Southern hemisphere was lower than the difference in the median value of MALR between Natural Forest and Plantations (8.9 vs. 12 g m^−2^ mon^−1^; Figure 2a). There was more variation in the distribution of mean monthly litterfall rates (MMLR) than MALR among grouped ecosystems (Figure 2b vs. a). MMLR had more unusually high values than MALR (Figure 2b vs. a).

**FIGURE 3:**
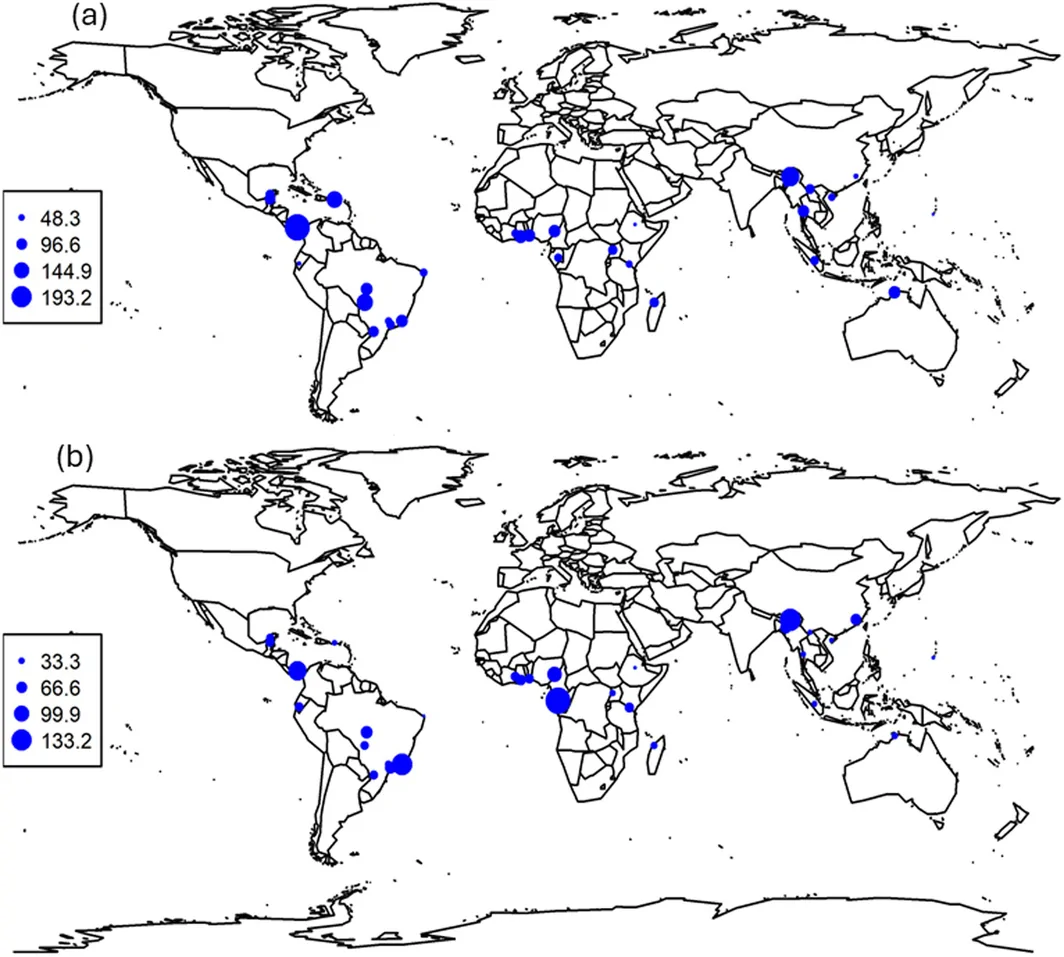
Map of seasonal mean leaf litterfall rates (g m^−2^ mon^−1^) for dry season (a) and wet season (b). The size of points indicates the litterfall rates.

Dry seasons mean litterfall rate was least across the African continent (Figure 3a) and highest across the Central and South American continents (Figure 3a). Wet seasons mean litterfall rate was similar across Central and South American continents and African continents (Figure 3b). Wet seasons mean litterfall was least across Asian and Oceanian continents (Figure 3b).

**FIGURE 4:**
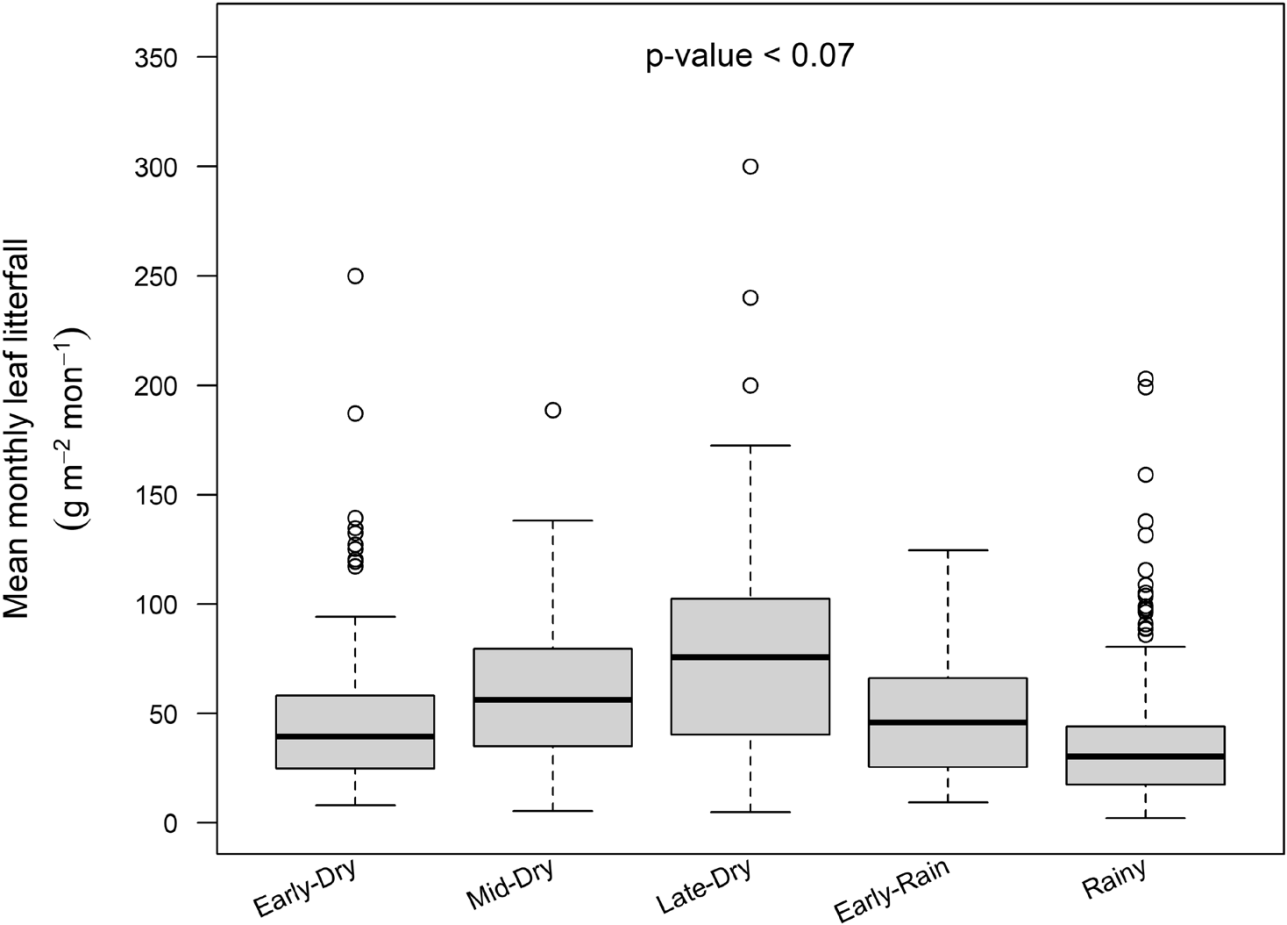
Seasonal distribution of monthly litterfall rates. The *p*‐value shown is the result of a two‐sample paired *t*‐test between “Mid‐Dry” and “Late‐Dry” datasets.

Across the data set, monthly litterfall rates varied considerably among early‐dry, mid‐dry, late‐dry, early‐rain, and rainy seasons (Figure 4). The largest variation in mean monthly litterfall rates across the data set was in the late‐dry season (Figure 4). Monthly litterfall rates were higher in the late‐dry season than the mid‐dry season (*p*‐value < 0.07, Figure 4), which partly supported hypothesis 1. Actually, the data provided marginal evidence about litterfall tending to peak later in the dry season.

**FIGURE 5:**
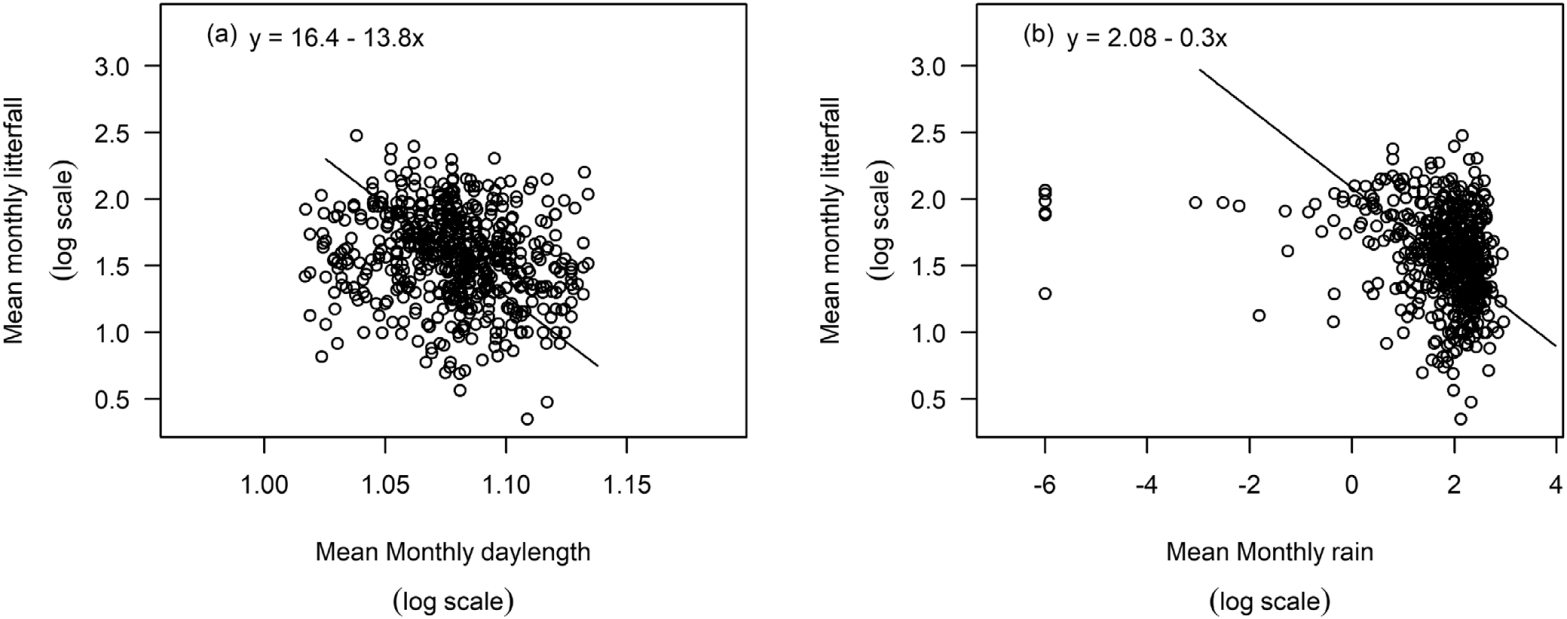
Relationship between monthly litterfall and monthly daylength (a) or between monthly litterfall and monthly rain (b). For monthly litterfall vs. monthly daylength; SMA slope = −13.8 (95% CI −14.95, −12.71), *r* ^2^ = 0.03 and *p*‐value < 0.01. For monthly litterfall vs. monthly rain; SMA slope = −0.3 (95% CI −0.32, −0.28), *r* ^2^ = 0.044 and *p*‐value < 0.01. The black line indicates the regression line, and the equation represents the equation of the regression line. Axes are log10 scaled.

Across the data set, mean monthly litterfall was negatively correlated with mean monthly daylength (Figure 5a). The strength of the correlation between litterfall and daylength was relatively weak but significant (*r* ^2^ = 0.03, *p*‐value < 0.01, Figure 5a) – making hypothesis 2 valid. Like in the case of daylength, the mean monthly litterfall had a negative correlation with rainfall (Figure 5b). The strength of the correlation between litterfall and rainfall was relatively weak too but significant (*r* ^2^ = 0.044, *p*‐value < 0.01, Figure 5b) – in support of hypothesis 3.

**FIGURE 6:**
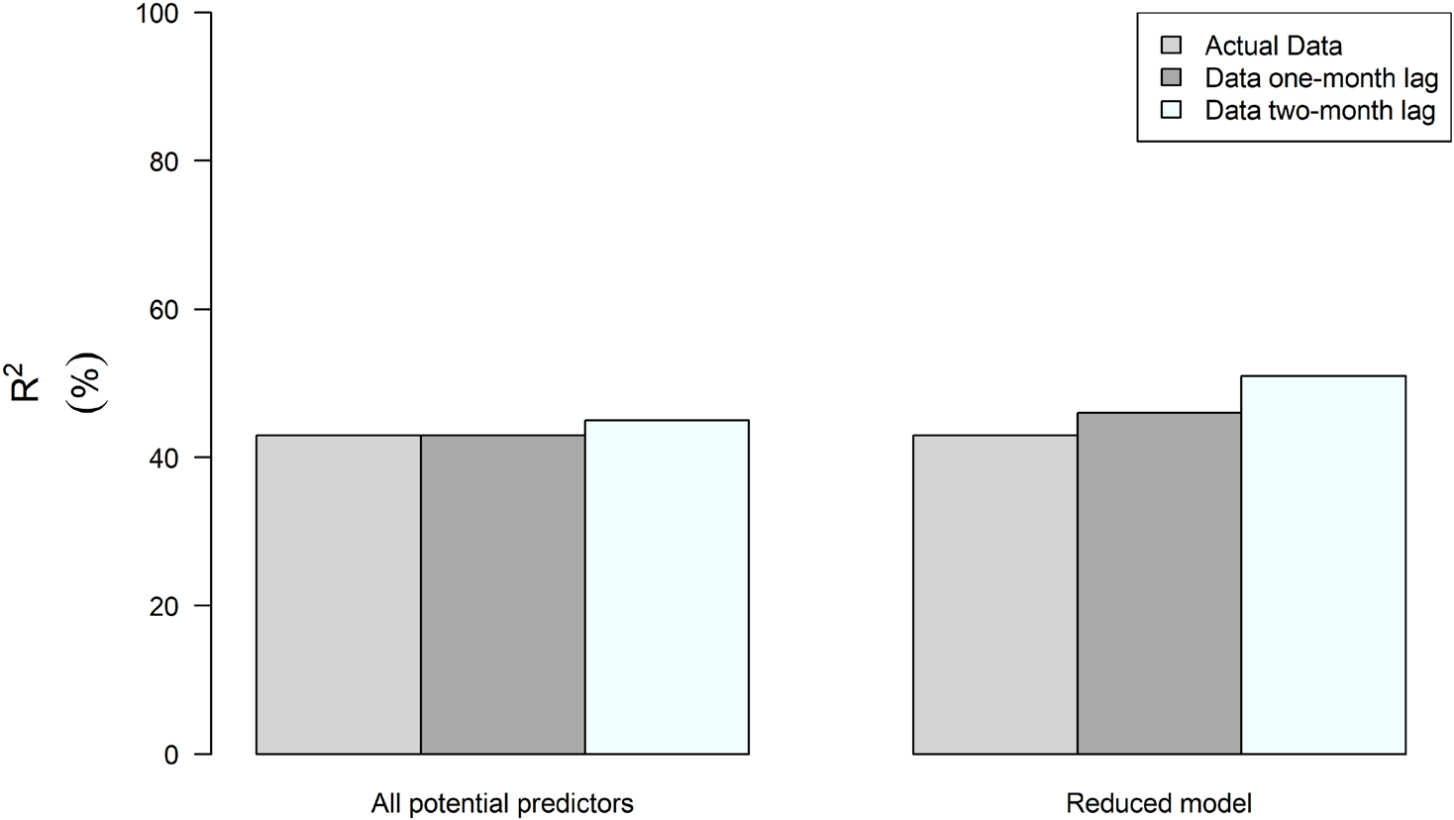
Coefficient of the determination (*R* ^2^) for the linear mixed effects model for different datasets (actual data, data 1‐month lag and data 2‐month lag) for (i) all potential predictors and (ii) final model (‘Reduced model’). The ‘all potential predictors’ refers to the LMEM that was obtained via the elimination method (LMEMe), and the ‘reduced model’ refers to reducing the predictors of LMEMe based on the significance of the *p*‐values and correlations among the fixed effects.

There was little difference in the explanatory power of the model when all potential predictors were used across the three different datasets; actual data, 1‐month lag data, and 2‐month lag data (Figure 6). The coefficient of determination (the *R* ^2^ values) ranged from 43% to 45%. When the predictors were reduced based on the significance of *p*‐values and correlation, there was a marked difference in the *R* ^2^ values, which ranged from 43% to 51% (Figure 6).

### 3.2 Lag Analysis, Climate and Edaphic Factor

The evaluation of model performance across different lag structures indicated that the 2‐month lagged climate structure provided the best fit for the data (Table 2). This model achieved the lowest Akaike information criterion (AIC = −14.51), demonstrating superior parsimony. It explained a substantial amount of variance, with a marginal *R* ^2^ of 0.25 for fixed effects and a conditional *R* ^2^ of 0.51 for the total model. The identity of the selected predictors for current‐month climate, 1‐month and 2‐month lag structures did not change in their respective reduced models. The model retained four predictors (radiation, daylength, rainfall, and soil pH), and successfully achieved convergence, confirming the stability of the estimates. The final model was based on 2‐month lagged predictors.

**TABLE 2:** Comparison of model performance and convergence across different lag structures, showing retained predictors, Akaike information criterion (AIC), marginal and conditional (*R* ^2^) values, and convergence status.

	Retained predictors	AIC	Marginal *R* ^2^	Conditional *R* ^2^	Model convergence
Current‐month climate	Radiation, daylength, rainfall and soil pH	21.14	0.17	0.43	No convergence failures
One‐month lagged climate	Radiation, daylength, rainfall and soil pH	11.70	0.20	0.46	No convergence failures
Two‐month lagged climate	Radiation, daylength, rainfall and soil pH	−14.51	0.25	0.51	No convergence failures

**FIGURE 7:**
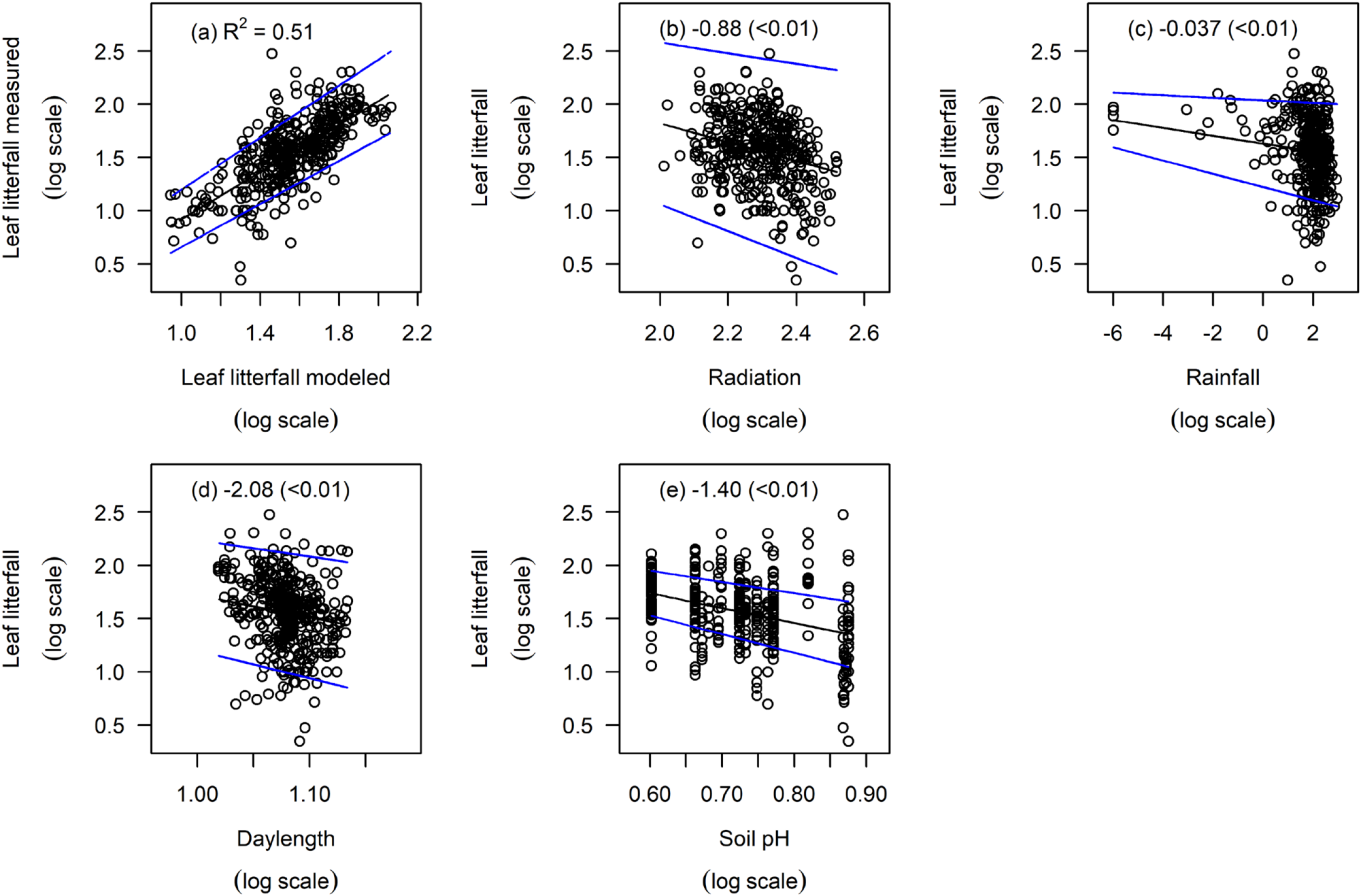
Relationship between measured litterfall and modeled litterfall (a), and relationships between monthly mean leaf litterfall and monthly climatic factors that include (b) radiation, (c) rainfall, (d) daylength, and (e) soil pH across the calibrated dataset. Each point corresponds to a monthly mean value. The solid line is the best fitted line. The overall *R* ^2^ value (a), coefficients, and *p*‐value of the regression (b–d) are also provided. The above results correspond to the linear mixed effects model that considered 2‐month lags of climate. Axes are log10 scaled.

Climate (radiation, rainfall and daylength) and edaphic factor (soil pH) explained 51% of the variation in measured litterfall rates across the calibration data set (Figure 7a). The *p*‐values of all of the predictors were generally low, indicating that these predictors were highly significant (Figure 7b–e). Litterfall rate was negatively correlated with all of the predictors (Figure 7b–e). Of the four predictors, daylength had the largest absolute coefficient (Figure 7d), indicating that litterfall in the dry season would be most sensitive to daylength.

Diagnostic tests of the LMEM showed that the variance of residuals is constant across fitted values (Figure S2a). The data points were randomly scattered around the zero horizontal line. The QQ‐plot showed that the residuals also followed a normal distribution, whereby the points mostly lay on the line (Figure S2b).

By performing multicollinearity checks, we found that the variance inflation factor (VIF; measures how much of an estimated regression coefficient is increased or inflated due to collinearity) is less than 1.21 (see Table 3). The value of VIF = 1 means no correlation. If VIF is between 1 and 5, then there is moderate correlation. Since VIF is slightly more than 1, the results indicate low correlation among the predictors, namely radiation, rainfall, daylength, and soil pH.

**TABLE 3:** Multicollinearity of the climatic and edaphic variables that were used as predictors of the model for leaf litterfall for all data.

	VIF	VIF 95% CI	Adj. VIF	Tolerance	Tolerance 95% CI
Radiation	1.13	[1.05, 1.33]	1.06	0.88	[0.75, 0.95]
Rainfall	1.15	[1.06, 1.34]	1.07	0.87	[0.74, 0.94]
Daylength	1.20	[1.10, 1.39]	1.10	0.83	[0.72, 0.91]
Soil pH	1.02	[1.00, 6.39]	1.01	0.98	[0.16, 1.00]

**FIGURE 8:**
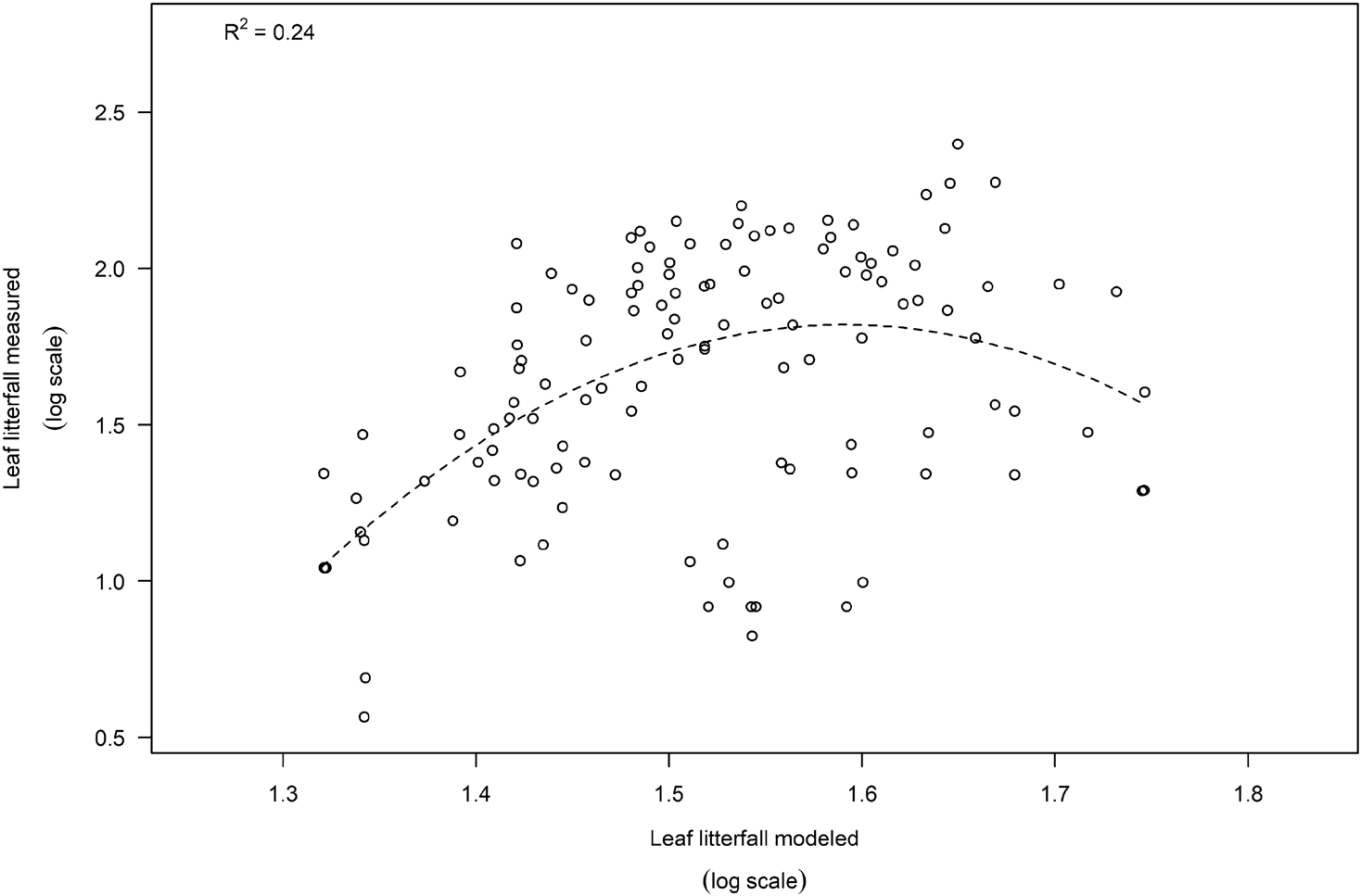
Relationship between measured litterfall and modeled litterfall across the validation sites. The dashed line indicates the best fitted line (polynomial) and the coefficient of determination is indicated by the *R* ^2^ value. The above results correspond to the linear mixed effects model that considered 2‐month lags of climate. Axes are log10 scaled.

When the derived model was applied to the validation data set, the explanatory power of the model was approximately halved (*r* ^2^ = 0.24; Figure 8). A large part of the variance for leaf litterfall in the validation data set was unexplained.

### 3.3 Random Forest

**FIGURE 9:**
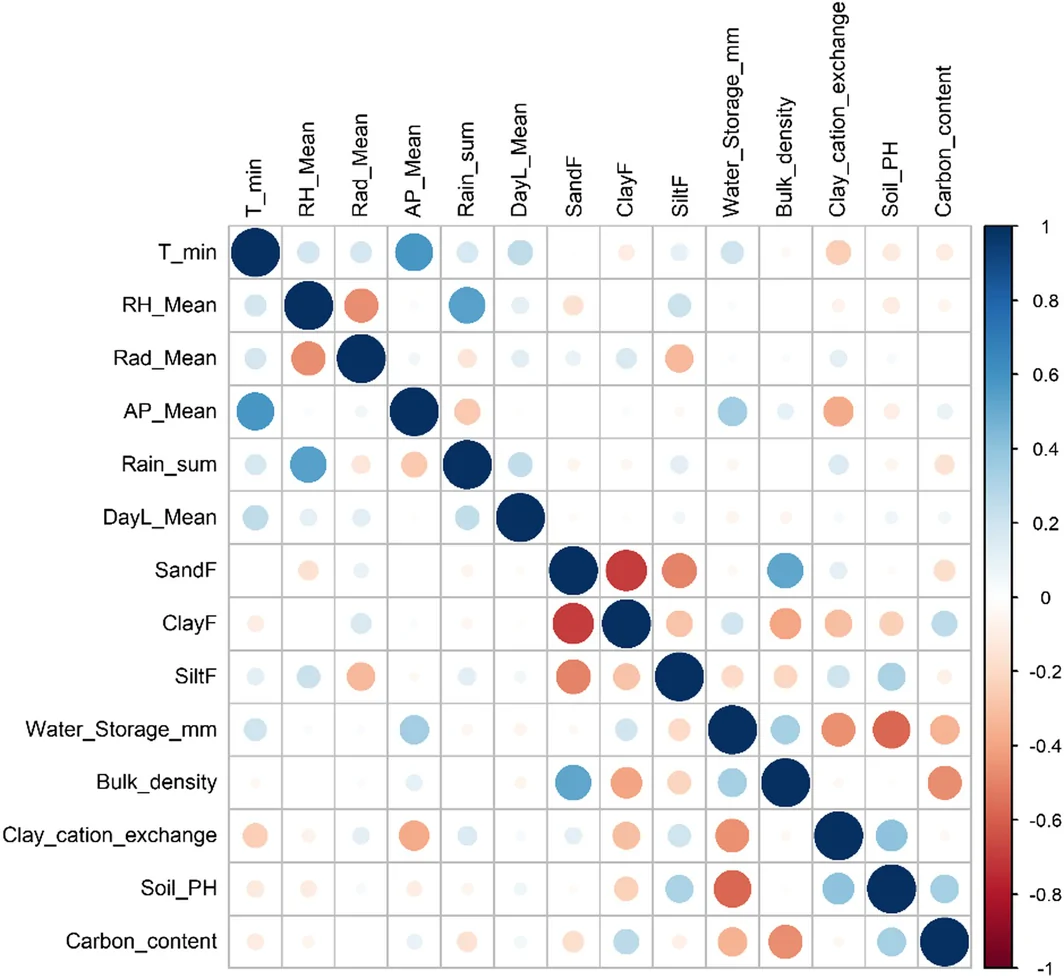
Pearson correlation between predictor variables for the random forest model. The definition of the variables is stated in Table S1.

Pearson correlation plot generally showed low correlation values among most of the predictor variables (Figure 9). This is not surprising because the highly correlated variables were filtered. Of the edaphic variables, sandF, clayF, soil pH, and water storage_mm tended to be correlated (Figure 9). In the case of climate variables, there was some apparent correlation between air pressure, minimum temperature, rainfall, and relative humidity (Figure 9).

**FIGURE 10:**
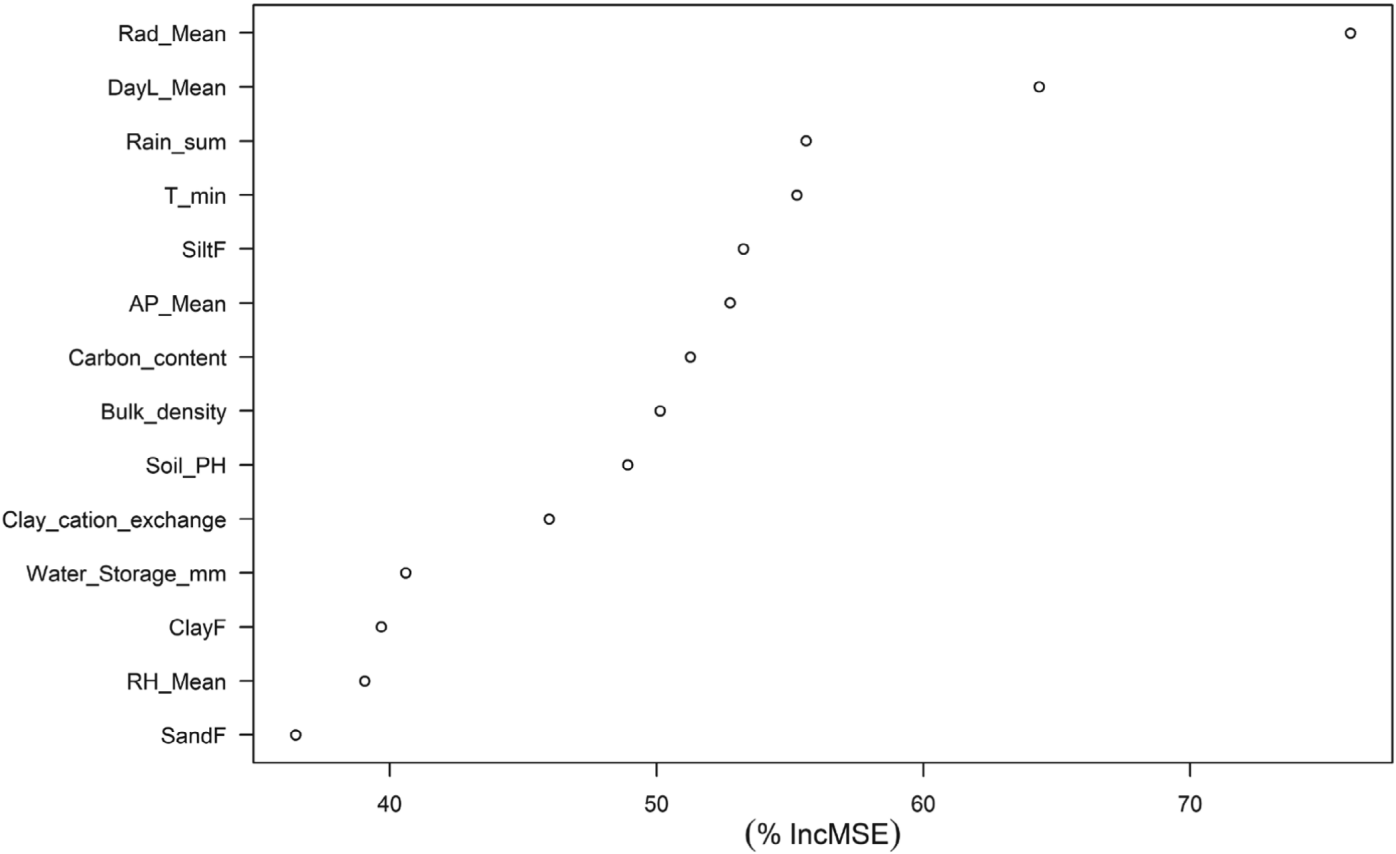
Outcome of the random forest model: Importance of the predictors based on Mean Decrease in Accuracy (%IncMSE). The *r* ^2^ value of the random forest was 47%. The definition of the predictors is stated in Table S1.

The predictors of the derived random forest model explained 47% of the variation in the measured leaf litterfall (Figure 10). Random forest model predicted radiation, daylength, and rainfall as the important predictors of the litterfall (Figure 10). These variables are most critical for the accuracy of the model. On the contrary, sandF, relative humidity, and clayF were the least important predictors of litterfall (Figure 10).

## 4 Discussion

### 4.1 Comparisons of Litterfall Rates of Forests With Plantations

We did not detect annual mean litterfall rates varying among all sites, northern hemisphere, southern hemisphere plantations, and natural forest ecosystems. Our results indicate that litterfall rates could be similar between natural forests and plantations. This contradicts earlier studies from China (Zhu et al. 2022) and from Indonesia (Kotowska et al. 2016), where both these groups of researchers found significantly lower litterfall rates in a monoculture rubber plantation compared to tropical natural forest. One of the reasons is that our database covers a wide range of forest types, exhibiting a large variability in litterfall. For instance, our database consists of litterfall data from the African continent, where at some sites there are two dry seasons in a year, one short and the other a longer dry season.

Annual leaf litterfall rates in a semi‐deciduous forest of Barro Colorado Nature Monument in Panama were 75 g m^−2^ mon^−1^ (Sayer and Tanner 2010), which is higher than the annual leaf litterfall for natural forests of our study sites. Higher litterfall rates in natural forests could be due to high tree density and canopy cover (Celentano et al. 2011). Another reason for higher litterfall rates in natural forest sites could be due to the presence of several species.

Our results indicated a marginal trend suggesting that litterfall tends to peak later in the dry season (*p* < 0.07). While this relationship did not meet the strict threshold for statistical significance (*α* = 0.05), the directional trend aligns with cumulative drought stress hypotheses. As the dry season progresses, cumulative water deficits typically trigger leaf senescence as a mechanism to minimize transpirational water loss. The weak statistical strength of this pattern in our data may stem from high climate variability across continents. Within dry month litterfall peaks have been documented in some studies (e.g., Tesfay et al. 2020), suggesting this marginal effect warrants further investigation via long‐term monitoring and likely high‐frequency sampling.

We found significant relationships between litterfall and daylength or litterfall and rainfall. As daylength decreases, litterfall rate increases, validating the second hypothesis. In this case, our study agrees with Borchert et al. (2002), who found decreasing daylength triggering leaf abscission. Photoperiod has been shown to be an important control over phenology in the tropics.

As rainfall decreases, litterfall rate increases—this result validated the third hypothesis and is in line with Aryal et al. (2015), however; Aryal's study was at a local scale. Chave et al. (2010) also found similar results to us at a large spatial scale when studying tropical forests from South America. The weak correlations between daylength and litterfall or between rainfall and litterfall indicate that there might be other factors acting as primary predictors of litterfall across semi‐deciduous ecosystems.

### 4.2 Predictors of Leaf Litterfall Rates

The statistical superiority of the 2‐month lagged climate structure over the current and 1‐month lag models highlights a distinct temporal decoupling between environmental predictors and forest canopy response. Rather than responding immediately to environmental conditions, the plants exhibit a biological delay in leaf shedding. This 2‐month buffer likely reflects the time required for physiological signaling or formation of leaf abscission layers following environmental stress. This finding suggests that integrating antecedent environmental conditions is critical for modeling and predicting carbon cycling and phenological shifts of tropical semideciduous ecosystems.

We found radiation, daylength, rainfall and soil pH associated with the litterfall. As radiation increases, litterfall decreases. One plausible reason why increasing radiation could decrease leaf shedding is through optimizing light use. Instead of dropping leaves, plants could be maximizing photosynthesis by replacing older and shaded leaves with new leaves (Wu et al. 2021). Another likely reason could be that plants are avoiding light limitation especially those that are in the dense sub‐canopy. To avoid getting shaded out plants flush new leaves to intercept the increased radiation thus leaf shedding is reduced (Martin et al. 2020).

As soil pH increases, litterfall rate decreases. One reason is that increasing soil pH could neutralize toxicity, enhance availability of vital macronutrients, and improve water and nutrient uptakes. Therefore, in such instances, leaf shedding could be reduced (Dhandapani et al. 2024).

### 4.3 Explanatory Power of the Model

About 51% of the variation in measured leaf litterfall rates was explained by the developed model. This is a moderate explanatory power of the model. However, this result is higher than that reported for tropical forests for South America, forexample, Chave et al. (2010) found rainfall explaining 10% of the variation in the litterfall. Chave et al. (2010) also could not detect associations of edaphic factors (e.g., soil type) with litterfall seasonality. We think the weak correlation could be due to the mixture of forest types in their study; that is, both evergreen and deciduous trees in the Chave et al. (2010) dataset.

Wang et al. (2016) investigated climatic factors associated with litterfall patterns of tropical and subtropical forests of Taiwan. Specifically, Wang and others acquired rainfall and land surface temperature from remote sensing and combined it with field litterfall estimates. They built a multiple regression model and found that temperature and precipitation explained 54% of the variation in litterfall rates. The LMEM's explanatory power in our study is similar to that of Wang et al. (2016).

As radiation, daylength, rainfall, and soil pH increase, litterfall decreases. A unit increase in daylength will decrease the litterfall the most. Soil pH is the second most important predictor. Elevated radiation and extended daylength may alter litter input dynamics, potentially influencing long‐term soil fertility and carbon storage.

The low explanatory power of the model across the validation sites (*r* ^2^ = 24%) indicates that the developed model is only moderately transferable across independent sites. That is, the model loses more than half of its ability to explain litterfall rates when applied to new sites. At the new sites, 76% of the variance is completely unexplained (residual error), indicating a high risk of cross‐site extrapolation. The polynomial fit clearly shows non‐linearity between measurements and model estimates. This could indicate a lack of biological processes in the derived empirical model. We think inclusion of functional traits such as leaf mass per area, leaf biomass, and leaf age could improve litterfall prediction. It is worth mentioning here that phenological models are generally poorly developed for tropical trees to date (Sakai 2001; Dahlin et al. 2015).

We would like to acknowledge that a single 75/25 split can be sensitive to which sites happen to be included in each subset and the validation performance can also depend on the split. To address the above issues, we performed a repeated site‐level cross‐validation. We used 26 regions per repeat and the same selected predictors and set the number of repeats to 10. The *r* ^2^ value for the validation was 26.7%. This indicates the model is stable across varying site configurations.

Random forest model analyses showed results consistent with the linear mixed effects model; the explanatory power of the two models was similar (*r* ^2^ = 47% vs. *r* ^2^ = 51%) and also both models highlighted radiation, daylength, and rainfall as the important predictors of the litterfall.

### 4.4 Unexplained Variation

There are three main factors that could potentially explain the unexplained variation in our measured litterfall: species composition and diversity, vegetation structure and functional traits, and soil nutrient availability. These mechanisms are not tested by the present study. More diverse communities may exhibit asynchronous leaf shedding, leading to more stable litterfall inputs over time, whereas less diverse or functionally similar communities may produce more synchronized pulses (Reich 1995; Giweta 2020). A high diversity of tree species in the forest ecosystem could increase organic carbon and C/N ratio in the soil (Vitousek and Sanford 1986). Diverse species in a forest can use different canopy positions in a way that results in more efficient partitioning of space and light resources (Sapijanskas et al. 2014), which can lead to increased ecosystem productivity and litterfall (Zheng et al. 2019).

Vegetation structure (e.g., basal area, canopy height, leaf biomass), functional traits (e.g., leaf lifespan, SLA), and disturbance history are all likely to influence litterfall too. For example, in a subtropical forest, a tree diversity experiment showed that higher stem densities and larger basal areas yielded greater total litter production (Wan et al. 2023). According to the leaf economic spectrum, ecosystems dominated by species that are fast‐growing and have high specific leaf area exhibit rapid leaf turnover and nutrient cycling (Wright et al. 2004). Disturbance history also affects litterfall rates. A study of forest plots from Singapore showed that low‐wood density species that were favored by increased light availability had to first overcome establishment barriers such as leaf litter in the storm‐affected sites, before being able to exploit the elevated gap resources (Lai et al. 2020).

A higher soil nutrient availability generally increases litterfall in tropical forests. Because tropical soils are often nutrient‐poor, the addition of key nutrients, especially phosphorus and nitrogen boosts plant growth and accelerates the turnover of leaves, resulting in a higher litterfall (Silver 1994). A recent study has shown that nutrient availability is associated withthe timing of leaf litterfall peak and leaf litter production (Manu et al. 2024).

We used a stepwise elimination method in our study. We acknowledge that this method has some weaknesses; it can produce unstable models and can inflate Type I error. It can also result in coefficient bias. All these can influence the statistical significance and distort the ecological interpretations.

## 5 Conclusion

We have examined data on leaf litterfall rates from different semi‐deciduous ecosystems. We were able to test hypotheses based on phenology and find out factors that determine litterfall rates. The results from our study suggest that climate (radiation, daylength, and rainfall) and edaphic factor (soil pH) together are partially explaining litterfall dynamics of ecosystem, indicating that the existing database can be extended by including other data sets as they become available. Our synthesized datasets can be used to calibrate litterfall rates that are predicted using process‐based models, such as land surface models.

## Author Contributions

**Ashehad A. Ali:** conceptualization (lead), data curation (supporting), formal analysis (supporting), investigation (equal), methodology (supporting), project administration (supporting), resources (equal), software (equal), supervision (supporting), validation (supporting), visualization (equal), writing – original draft (equal), writing – review and editing (equal). **Anuj T. Magar:** conceptualization (supporting), data curation (lead), formal analysis (equal), investigation (equal), methodology (lead), resources (equal), software (lead), writing – original draft (lead), writing – review and editing (supporting).

## Funding

The authors have nothing to report.

## Conflicts of Interest

The authors declare no conflicts of interest.

## Supporting information


**Table S1:** Climate, soil and vegetation structure related variables, their definitions and units as listed in the Excel spreadsheet.
**Table S2:** Number of study sites available for each vegetation variable.
**Figure S1:** Map indicating the dataset used for developing the model (blue points) and validating the model (green points).
**Figure S2:** Residual diagnostic test plot (a) and Q‐Q density plot of the final linear mixed effects model for the calibrated case. The above results correspond to the linear mixed effects model that considered 2‐month lags of climate.

**Data S1:** Supporting Information.

## Data Availability

The data used in this study are provided as a Supporting Information.
