# Supplementary material for: Climate and Edaphic Factors Partly Explain Leaf Litterfall Rates of Species From Tropical Semi‐Deciduous Ecosystems
Source: Ecol Evol. 2026 Aug 20;16(8):e74224. doi: 10.1002/ece3.74224 (PMC13491141; doi:10.1002/ece3.74224)
Supplement: Supplementary file 1 [file ECE3-16-e74224-s001.docx]

**Appendix**

**Tables**

| **Variable** | **Definition** | **Unit** |
| --- | --- | --- |
| Site | name of the site | unitless |
| latitude | geographic coordinate | decimal |
| longitude | geographic coordinate | decimal |
| Year | measurement year | unitless |
| month | measurement months | unitless |
| season | measurement season (1=Dry and 2=Wet) | unitless |
| doystart | day start for the beginning of the month | unitless |
| doyend | day end for the ending of the month | unitless |
| T_Mean | monthly mean air temperature 2m | °C |
| T_max | monthly maximum air temperature 2m | °C |
| T_min | monthly minimum air temperature 2m | °C |
| RH_Mean | monthly mean relaitve humidity2m | unitless |
| RH_max | monthly maximum relative humidity 2m | unitless |
| RH_min | monthly minimum relative humidity 2m | unitless |
| VPD_Mean | monthly mean vapor pressure deficit 2m | kPa |
| VPD_max | monthly maximum vapor pressure deficit 2m | kPa |
| VPD_min | monthly minimum vapor pressure deficit 2m | kPa |
| Rad_Mean | monthly mean global radiation 2m | W m^-2^ |
| Rain_sum | monthly maximum global radiation 2m | W m^-2^ |
| AP_Mean | monthly minimum global radiation 2m | W m^-2^ |
| DayL_Mean | monthly mean day length | hours |
| WS_Mean | monthly mean wind speed | m s^-1^ |
| litterfall | monthly leaf litter | g DM m^-2^ mon^-1^ |
| SandF | Top soil sand fraction | % weight |
| ClayF | Top soil clay fraction | % weight |
| SiltF | Top soil silt fraction | % weight |
| water_storage | soil water storage capacity | unitless; capacity decreases as values increase |
| water_storage_mm | mm water per m of soil unit | mm |
| Bulk_density | Top soil bulk density | kg dm^-3^ |
| Clay_cation_exchange | Cation exchange capacity of clay in subsoil | cmol per kg |
| Soil_PH | Topsoil pH | unitless |
| Carbon_content | Topsoil carbon content | kg C m^-2^ |
| Soil_Organic_Carbon | Soil organic carbon content | Kg C m^-2^ |
| elevation | elevation of the site | m |
| Hemisphere | Hemisphere (nothern or southern) | unitless |
| Ecosystem_type | native or plantation | unitless |
| Country | country where the site is located | unitless |
| Dry_season | (1=early dry season, 2=mid dry season, 3=late dry season, 4=early rain, 0 = rainy) | unitless |
| DBH | diameter at breast height | cm |
| Stand_density | stand density | trees ha^-1^ |
| Basal_area | basal area | m^2^ ha^-1^ |
| Canopy_height | canopy height | m |
| Leaf_biomass | leaf biomass | kg ha^-1^ |
| LMA | leaf mass per area | g m^-2^ |
| Region | region in which sites are located | unitless |

**Table A1** Climate, soil and vegetation structure related variables, their definitions and units as listed in the Excel spreadsheet.

| **Biotic Variables** | **Number of Sites** |
| --- | --- |
| Stand density | 14 |
| Basal Area | 9 |
| Canopy Height | 7 |
| Leaf biomass | 1 |
| DBH | 12 |
| LMA | 2 |

**Table A2** Number of study sites available for each vegetation variable.

**Figures**

**Figure A1** Map indicating the dataset used for developing the model (blue points) and validating the model (green points).

**Figure A2** Residual diagnostic test plot (a) and Q-Q density plot of the final linear mixed effects model for the calibrated case. The above results correspond to the linear mixed effects model that considered two-month lags of climate.
